# Null-model-based network comparison reveals core associations

**DOI:** 10.1038/s43705-021-00036-w

**Published:** 2021-07-16

**Authors:** Lisa Röttjers, Doris Vandeputte, Jeroen Raes, Karoline Faust

**Affiliations:** 1 Laboratory of Molecular Bacteriology, Rega Institute, KU Leuven, Leuven, Belgium; 2 VIB-KU Leuven Center for Microbiology, Leuven, Belgium

**Keywords:** Microbiology, Ecology

## Abstract

Microbial network construction and analysis is an important tool in microbial ecology. Such networks are often constructed from statistically inferred associations and may not represent ecological interactions. Hence, microbial association networks are error prone and do not necessarily reflect true community structure. We have developed anuran, a toolbox for investigation of noisy networks with null models. Such models allow researchers to generate data under the null hypothesis that all associations are random, supporting identification of nonrandom patterns in groups of association networks. This toolbox compares multiple networks to identify conserved subsets (core association networks, CANs) and other network properties that are shared across all networks. We apply anuran to a time series of fecal samples from 20 women to demonstrate the existence of CANs in a subset of the sampled individuals. Moreover, we use data from the Global Sponge Project to demonstrate that orders of sponges have a larger CAN than expected at random. In conclusion, this toolbox is a resource for investigators wanting to compare microbial networks across conditions, time series, gradients, or hosts.

## Introduction

A biologically interesting pattern needs to differ from patterns observed by chance or from patterns generated by processes that are not of interest to the investigator [1]. Such differences can often only be observed by generating data under the sets of rules specified by null models. For network inference, tools such as CoNet and LSA use a conceptually simple null model where shuffled data are presumed to represent the situation without meaningful biological structure [2, 3]. In the analysis of microbial networks however, null models are not yet systematically employed. As a result, network properties may incorrectly be presumed to reflect a characteristic of interest, when they result directly from properties of the count table. For example, differences in clustering coefficients could result from imbalanced sample numbers. Here, we present a tool for generation of randomized networks through null models that retain important characteristics of the original networks.

Microbial association networks have been shown to be inaccurate on simulated data [4–6]. There are multiple reasons for this, such as the appearance of indirect edges when biotic or abiotic factors are not included in network inference [6, 7]. In addition, technical noise (introduced via sequencing methods) affects network inference [4]. Moreover, ecological processes such as higher-order interactions are ignored in pairwise association inference. Microbial sequencing is rarely able to resolve the spatial resolution of microbial interactions, which are often limited to a range of a few micrometers [8]. Finally, sample numbers are often insufficient to infer associations accurately.

In this context, null models can help identify network properties that are different from what is expected based on the null hypothesis that networks are mostly random. One of those properties of interest is the edge intersection of networks, the group of edges present in a combination of networks. Comparisons across multiple networks have previously been used to identify meaningful associations across populations. For example, networks inferred with different methods have been compared to identify meaningful associations [9]. Similarly, networks inferred from geographically separated human populations were shown to be more similar than expected by chance [10].

For such comparisons, null models are crucial to identify similarities across networks not driven by similarity in species composition. The edge intersection of networks can become large even for completely random networks due to similarities in the abundance data. These similarities are relevant when ecosystems consist of a number of “core” genera that are found in most samples of the ecosystem. Such core microbiomes have been identified for the human gut [11], the oral microbiome [12], and the coral microbiome [13]. Yet, presence of a core microbiome does not necessarily imply presence of a core microbial interaction network, since the same microorganisms may interact in different ways, fluctuations of their abundances may not be driven by interactions, or the data may be too noisy to infer associations. This raises the question of whether interactions are preserved across different realizations of an ecosystem, whether they are preserved within subgroups of the ecosystem, or whether they are unique [14]. Since null model analysis can distinguish random from significant intersection sizes, it can test for the presence of one or more core association networks (CANs) within a group of association networks. Core associations can then be explored to check whether they represent ecological interactions. Hence, null model analysis is a step toward answering whether interactions are universal.

Prior work demonstrated that many network properties, specifically centralities, can be highly correlated, but may represent different underlying aspects in network topology [15]. Null models were implemented in the bipartite package to demonstrate that the degree and edge density were strongly correlated to species number, while the cluster coefficient and connectance increased as the number of observed edges per species (sampling intensity) increased [16]. The BiMat MATLAB package uses a similar null model strategy to estimate significance of network properties including nestedness, modularity, and module structure [17]. Chung-Lu and Erdős-Rényi null models have also been applied to study average path length, modularity, diameter, and clustering coefficients [18]. In each of these studies, a network property of interest could be interpreted meaningfully after comparison to random networks. In contrast to studying one network in particular, our null model strategy addresses the existence of conserved associations. While applications for network analysis such as NetConfer and setsApp also return intersections of networks [19, 20], these applications do not assess whether this intersection is nonrandom.

**Fig. 1:**
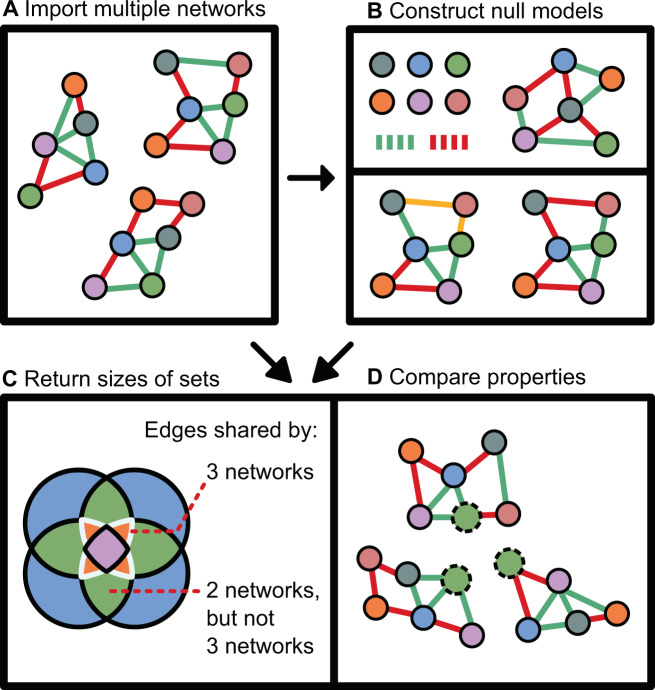
The anuran pipeline. In the networks, node colors represent microbial taxa, while the red and green edge colors represent negative and positive edge weights respectively. **A** Multiple networks are imported by the user. These networks can be ordered, and multiple groups of networks can be imported at the same time. **B** Random networks are constructed for each of the imported networks. These can be fully randomized by removing all edges and reassigning them randomly. Alternatively, they can preserve the degree distribution of the original network by swapping edges (highlighted in yellow). They may also contain a synthetic core. **C** A Venn diagram showing the types of sets returned by the toolbox for a collection of four networks. These sets measure the overlap between specific numbers of networks; each color in the Venn diagram indicates a set returned by the toolbox. Rather than returning the matching edges between two specific networks, the toolbox returns any matching edges that are present in at least three networks (intersection) or only present in two networks (difference of two intersections), as indicated by the different colors in the Venn diagram. **D** Node properties (such as the degree of the green taxon with dashed border) can be compared to degree distribution of this node in randomized networks to assess whether this taxon has a nonrandom degree centrality across networks. Similarly, network properties can be compared to those calculated for random networks.

In this manuscript, we introduce a null model strategy based on shuffled networks, where relationships between taxa are randomly reassigned (Fig. 1). We illustrate the power of this strategy by identifying nonrandom CANs in human gut and sponge microbial networks and show that the CANs represent biologically relevant group-specific associations. These strategies have been implemented in a software toolbox that evaluates the significance of network or network property comparisons.

## Materials and methods

### Null model toolbox

We have developed a software toolbox, anuran, (**a** toolbox with **nu**ll models for identification of non**r**andom patterns in **a**ssociation **n**etworks) that generates random networks and assesses properties of these networks. Three types of networks can be generated in the current implementation: completely randomized networks, degree-preserving networks, and a variation of both networks that keeps a fraction of the edges fixed. Networks without a synthetic CAN, meaning they do not contain any fixed edges, are referred to as negative controls in the remainder of the manuscript, while networks with a synthetic CAN are referred to as positive control networks. In combination, these null models can generate CAN sizes for (1) the situation where all edges are entirely random, (2) the situation where taxa connecting edges are random, but the presence of an edge is not, and (3) the situation where part of a network is random but the remainder is part of a CAN.

For the completely randomized model, a network is initialized with the same nodes as the input network. Edges are then added randomly until the total edge number is equal to the number of edges in the input network. For the degree-preserving model, edges are swapped rather than removed and added back to the network, so that two edges (a, b) and (c, d) become the new edges (a, c) and (b, d). Hence, the model preserves the degree distribution found in the input network and each node has the same degree as it has in the original network, but other centralities such as the betweenness centrality can change. The user specifies both the number of random networks generated for each network (by default 10) and the number of sets (collections) of these networks (by default 50) that are sampled to calculate set sizes.

As stated previously, variations of the above two null models can be used to construct positive control networks. For this procedure, a fraction of edges is extracted from the total union of edges across all networks. For fully randomized networks, these edges are first added, then edges are added until the total number of edges in the original network is reached. For the degree-preserving randomized networks, negative control networks (with preserved degree) are first generated. Then, for each edge in the fixed core, the algorithm attempts to find two edges that can be swapped so the fixed edge is created. If this fails, a random edge is deleted and the fixed edge is introduced, so the degree is not exactly preserved. To swap the edges successfully, it is necessary that each of the nodes participating in a fixed edge has another edge not part of the fixed core. As a result, the degree distribution can change significantly for networks where nodes in the fixed core are disconnected or where the fixed core is very large compared to the positive control network.

It is possible to include nodes without significant associations in the network file as disconnected nodes (orphan nodes) by supplying the network file with the orphan nodes included as nodes without any edges. In this case, the random model reflects a situation where associations are randomly selected from all taxa. However, the degree-preserving networks are not affected by orphan nodes. The inclusion of orphan nodes leads to different estimates for set sizes for the random model that may lead to an overestimation of the significance of a CAN, as most taxa are too rare to acquire associations. Therefore, we ignored the presence of disconnected nodes in our case study.

The toolbox has been implemented in Python 3.6 and consists of both an application programming interface and command-line interface (CLI). Documentation for the toolbox has been included as a supplement (Supplementary File 1), with this and additional vignettes available through the GitHub page at https://github.com/ramellose/anuran. Currently, the CLI pipeline assesses set sizes, (rank-transformed) betweenness, degree, and closeness centrality scores and several network-level properties: degree assortativity, connectivity, diameter, radius, and average shortest path length (Fig. 1). NetworkX implementations of these centrality calculations were used [21].

The software uses a set-of-sets approach to identify CANs. A set is a specific collection of edges, such as the intersection set, which is the collection of edges present across multiple networks. The CANs are identified as differences of specific intersection sets. Hence, the toolbox specifically identifies sets and sets of sets that are likely to be of interest for microbial association networks. These sets represent collections of edges that are only present in one specific fraction of networks and distinguish between less conserved and more conserved edges.

An example with four networks is illustrated with a Venn diagram (Fig. 1c). To obtain the difference of the intersections, the set that includes one or more additional networks is subtracted from the intersection set that includes fewer networks. These sets are referred to as combinations of intersections with fractions or integers, i.e., the intersection 0.5 refers to all intersections of 50% of the networks. Similarly, set of sets are identified by a combination of intersection numbers: the set of sets 6→10 refers to the difference of intersection 6 and intersection 10 and therefore contains no edges present in at least 10 networks. For most analyses, the difference of intersections is preferred over intersections since the intersections are nested. By taking the difference, it is possible to distinguish between more and less conserved associations.

The equations for differences and *k*-intersections for groups of *n* networks are given below. The equations only refer to edge sets *E*, so they do not apply to numbers of matching nodes. The difference is the union of all sets *D*_*i*_ for 1 up to *n* networks, where the sets *D*_*i*_ contain all edges *x* present in an edge set *E*_*i*_ but not in the union of all other edge sets

$${{{\mathrm{Difference}}}} = \mathop {\bigcup}\limits_{i = 1}^n {D_i} \;{{{\mathrm{where}}}}\;D_i = \left\{ {x:x \in E_i,x \,\notin\, \mathop {\bigcup}\limits_{\begin{array}{*{20}{c}} {j = 1} \\ {i \ne j} \end{array}}^n {E_j} } \right\}$$

The *k*-intersections are unions of intersections *S*_*I*_. These intersections *S*_*I*_ are sets of groups of edge sets, where the groups *I* are *k*-permutations of *n* and *E*_*i*_ is a single edge set in *I*. Hence, for a total number of edge sets *n*, each of the groups *I* have size *k* and the collection of all possible groups is indicated as $P_k^n$. For the 4-intersection for a group of 40 edge sets, the size of $P_k^n$ can be calculated as the binomial coefficient $$\Big( {\begin{array}{*{20}{c}} {40} \\ 4 \end{array}} \Big)$$. This mathematical representation is not implemented directly in the software, as the software simply takes the set of all edges present in at least four networks and therefore ignores network identity.

Hence, a *k*-intersection is the union of all intersections *S*_*I*_ for *I* in $P_k^n$

$${{{\mathrm{Intersection}}}} = \mathop {\bigcup}\limits_{I \in P_k^n} {S_I} \;{{{\mathrm{where}}}}\;S_I = \left\{ {x:x \in \mathop {\bigcap}\limits_{i \in I} {E_i} } \right\}$$

Since edges present in at least *k* networks but not in *m* networks represent less conserved edges, the difference of the intersections is calculated to distinguish between less conserved and more conserved edges. The difference of two intersections *k* and *m*, with *S*_*I*_ and *S*_*J*_ defined identically to *S*_*I*_ in the equation above is then given below

$${{{\mathrm{Difference}}}}\;{{{\mathrm{of}}}}\;{{{\mathrm{intersections}}}} = \mathop {\bigcup}\limits_{I \in P_k^n} {S_I} \backslash \mathop {\bigcup}\limits_{J \in P_m^n} {S_J} \;{{{\mathrm{where}}}}\;k < m$$

To compare observed set sizes to set sizes of random networks, the *Z*-score test is carried out, which identifies set sizes in the input networks that are outside the range of set sizes inferred from groups of random networks. The SciPy normaltest implementation [22] of D’Agostino’s and Pearson’s omnibus normality test is used to test for both kurtosis and skewness [23, 24]. Since this test requires at least 20 observations, a warning is issued if the number of random networks needs to be increased.

The toolbox can also assess centrality scores across networks. To ensure that centralities are not biased by edge number, these are first converted to ranks before a Mann–Whitney *U* test is used to assess whether the distributions of ranks are similar across groups of observed networks and random networks. The comparisons to random networks are repeated a number of times and parameter-free *p* values across all comparisons are calculated from the number of successful Mann–Whitney *U* tests. By default, Benjamini-Hochberg multiple-testing corrections (implemented in the statsmodel package) are carried out on these *p* values to correct for the number of taxa [25]. The approach for network-level properties is similar, with the software currently supporting assortativity, connectivity, diameter, radius, and the average shortest path length. If the networks are ordered, the toolbox can calculate Spearman correlations of these properties to the network order. For example, users could supply networks constructed across a pH gradient. The results of all analyses are exported to tab-delimited files so they can be further analyzed and visualized in the user’s preferred statistical environment.

Finally, the toolbox includes an option for resampling networks. In this way, the resulting data show how trends in set sizes change as the number of networks is increased. The resulting data can be interpreted as a rarefaction curve, where flattening of the curve suggests that sufficient networks have been collected to identify all edges present in a specific fraction of networks.

### Case studies

Gut microbial time series data were collected from 20 women each of whom donated stool samples for over a month, with a sampling frequency close to one sample per day (Vandeputte et al., submitted) [26]. These women also reported data on their menstrual cycle. For each sample, enterotype assignments were carried out as in Vandeputte et al. [27] with Dirichlet multinomial clustering. Samples were assigned to Bacteroides 1, Bacteroides 2, Ruminococcaceae, or Prevotella.

Progression through the menstrual cycle was rescaled to 28 days (the average length of a menstrual cycle) for all women. For days where there was more than one sample, only the first sample was used. Taxa present in less than 50% of participants were discarded from the analysis. Association networks were constructed with fastLSA v1.0 [28] with data rarefied to 10,000 sequences per sample, with correlations inferred across a delay of three time points (*α* = 0.05). Set sizes were analyzed with anuran, by generating 20 networks per observed network and resampling 100 different groups from these. Positive controls were generated 20 times, with a core size equal to 20% of the union of edges at 10% prevalence (edges present in at least two networks) and at 50% prevalence (edges present in at least ten networks). Set sizes and centralities with a *p* value below 0.05 for comparisons to values from random networks were considered significantly different from the random networks. The anuran toolbox was also used to assess the effect of increasing the number of participants.

The Walktrap community finding algorithm [29], implemented in the igraph R package v1.2.6 [30], was used to cluster the inferred CAN as the lack of negative edges in the CAN suggested that random walks could sufficiently identify clusters. To visualize enterotype-specific patterns of relative abundance, we computed the mean relative abundance of taxa per individual. We then took the median relative abundances across all individuals who belonged predominantly to the Ruminococcaceae enterotype, an enterotype previously linked to lower stool moisture [27], and subtracted from these all other median relative abundances, giving an estimate of taxa that had high abundance in the Ruminococcaceae enterotype compared to other enterotypes.

For the case study on the sponge microbiome, QIIME-processed data were downloaded from Moitinho et al. [31]. Samples with fewer than 1000 counts were removed and the samples were rarefied to even depth at 1034 sequences. After rarefaction, the abundance data were first filtered for 20% taxon prevalence across all samples, then once more to ensure 20% prevalence across different orders. Counts for removed taxa were retained to preserve the sample sums. After excluding host orders with fewer than 50 samples, 10 orders remained. CoNet v1.1.1 with renormalisation was then used to infer association networks (Faust and Raes [2]). Edges were generated with Pearson correlation, Spearman correlation, mutual information, Bray–Curtis dissimilarity, and Kullback–Leibler distance. Edges were included if at least one method reached significance; only edges with a combined *Q*-value below 0.05 (estimated using a combination of permutation and bootstrapping) were retained. The CoNet CANs were inferred with anuran generating 20 negative control random networks per host order and resampling these 100 times. For the positive controls, 20 network groups were generated with a core size equal to 20% of the union of edges at 20% prevalence (edges present in at least two networks) and at 50% prevalence (edges present in at least five networks). Set sizes and centralities with a *p* value below 0.05 for comparisons to values from random networks were considered significantly different from the random networks. CoNet networks were compared to FlashWeave networks [7]. FlashWeave v0.16.0 was run as FlashWeave-S (sensitive set to true and heterogeneous to false), with all other settings set to the default. To compare FlashWeave networks to CoNet networks, anuran generated five randomized networks per order-specific network and resampled these five times.

Prior research indicated that microbial abundance was a significant driver of community structure in sponges [32]. Therefore, taxa in the CAN were compared to taxa reported as indicators of high microbial abundance (HMA) or low microbial abundance (LMA) [32]. CAN network clusters were identified with manta v1.0.0 [33], as this algorithm has been designed to handle negative edges in the CAN. To run the clustering algorithm, default settings were used, except the number of iterations and permutations, which was set to 200. A Chi-squared test was used to compare HMA–LMA predictions to CAN cluster assignments (*α* = 0.05).

## Results

### Null models support the existence of a small CAN in the human gut

We inferred 20 networks from time series of stool samples with the fastLSA network inference method [28], one network per person. The median edge number of these networks was 35.5, but one network contained only 6 edges while another contained 294 edges, indicating that there was significant variability in edge number. Despite these differences, anuran was able to identify relevant patterns through the use of null models and reported that a low-prevalence CAN exists, with associations found in 20–25% of individuals.

**Fig. 2:**
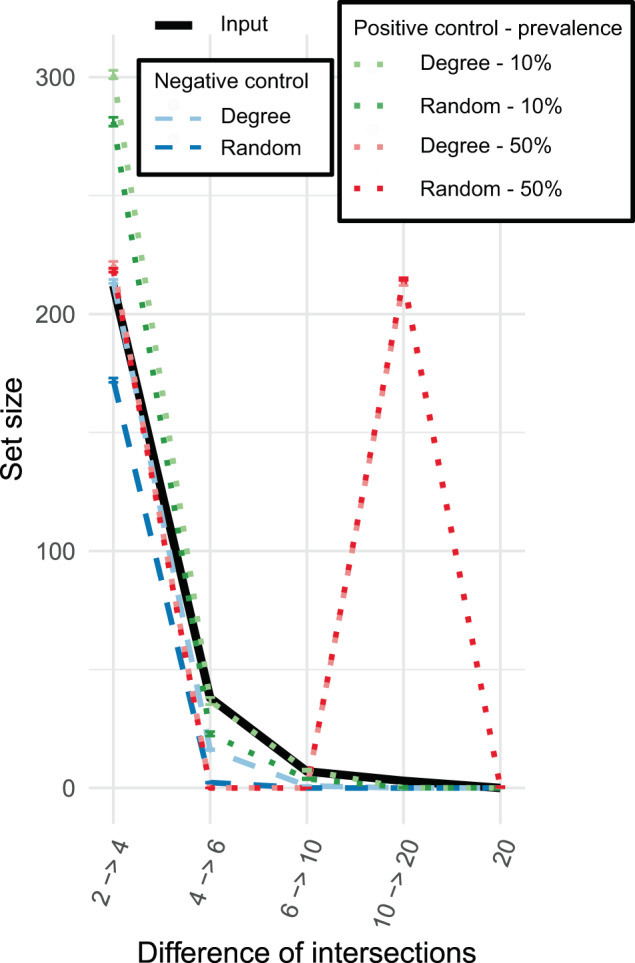
Set sizes across networks for 20 host-specific human gut networks and randomized networks. The set size is the number of edges present in a particular number of networks. The set size is shown for a set of sets of network intersections, meaning that the CAN 4→6 is calculated as the number of edges in four or five networks with all edges in at least six networks removed. Each input network was generated from stool samples collected from healthy volunteers and is built for a single volunteer. These networks were then randomized either with the same degree distribution (degree) or without this distribution (random). Moreover, each of the networks was randomized with preservation of a part of the network union for a subset of the randomizations as a positive control. Hence, the positive control degree networks are randomized versions of the group of observed networks with 20% of the inferred associations present in at least 10% of individuals or in at least 50% of individuals. Error bars represent the standard error across different combinations of random networks. For edges present in 4–6 networks, the set size of the input networks deviates significantly from the set size from those of random networks with or without degree preservation.

The intersection of the observed networks in contrast to the intersection of negative control networks supports the existence of a small CAN (Fig. 2). The CAN 4→6 (associations present in at least four networks but not in six networks) was much larger compared to the negative control networks, with the CAN containing 38 associations versus a median of 2 associations and a median of 16 associations for the fully randomized and degree-preserving negative control networks, respectively. Even the CAN for the positive control networks with 10% edge prevalence was slightly smaller, at 22 and 36 associations for randomized and degree-preserving positive control networks, respectively. The anuran-reported *p* values (*Z*-score test) confirm the observed trends; the set sizes of all but one tested difference of intersections (differences up to 0.5) are different when comparing the input networks to the negative control networks (*p* < 0.0001). Only the set size of the two-network difference is not significantly different compared to the degree-preserving negative control networks (*p* = 0.17). Consequently, we could not identify whether there were more associations conserved between only two participants than we expect from the degree distribution alone.

Only three associations occur in ten or more networks. Hence, we concluded that there is a low-prevalence CAN, but there are no associations that are conserved across most or even half of the individuals. Moreover, the resampling analysis demonstrates that the number of networks is insufficient to identify the size and prevalence of the CAN (Additional File 1: Fig. S1). A simulation shows that both difference and intersection should stabilize after a certain number of networks (Additional File 1: Fig. S2), but this is not observed for the resampling analysis. The simulation suggests that 30–40 networks would be necessary to find associations present in 33% of networks.

Only four taxa, which were assigned to *Dorea*, *Blautia*, Clostridiales, and Ruminococcaceae, had an uncorrected *p* value below 0.15 for any of the permutation tests comparing the distributions of degree, betweenness or closeness centralities (Additional File 1: Fig. S3). Low *p* values were not found for comparisons to degree-preserving negative control networks, suggesting that degree distribution alone can sufficiently explain high centrality rankings.

**Fig. 3:**
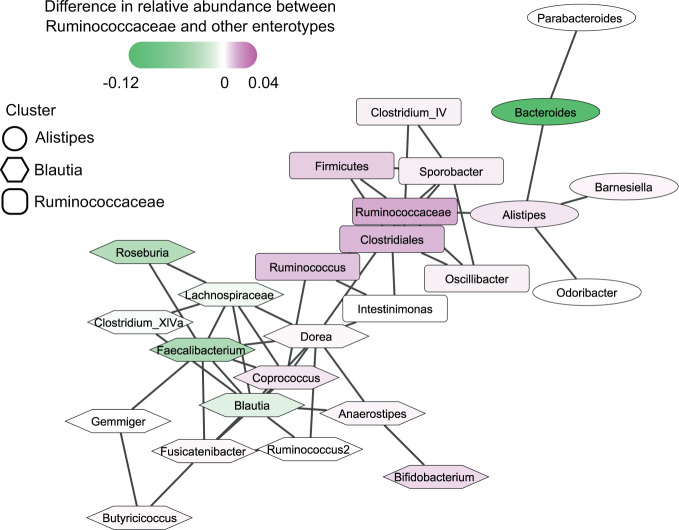
Core association network (CAN) constructed from associations present in at least four participants. Associations present in at least four networks were included in the CAN, which was clustered with the Walktrap method for community detection. Clusters are named after the most central taxa. Node color is mapped to the difference between median relative abundances for the Ruminococcaceae enterotype compared to other enterotypes. Therefore, the pink color indicates that a taxon was more abundant in the Ruminococcaceae enterotype, while a green color suggests that the taxon was less abundant in that enterotype. Node labels for higher taxonomic levels indicate that the taxon is an unclassified member of a taxonomic group. All edge weights were positive.

As the intersection of four participants was larger than expected from the negative control networks, the CAN from this intersection was further investigated (Fig. 3). The CAN was divided in three clusters with the Walktrap algorithm [29]. Of the two larger clusters, one contains *Dorea*, *Blautia*, and *Faecalibacterium* as its highest-degree nodes, while the other contains *Sporobacter*, a group of Ruminococcaceae members, and a group of Clostridiales members as its highest-degree nodes. Clusters were named after their most central nodes. Because enterotypes were not equally distributed across individuals, we could not carry out any statistics to connect network clusters to enterotypes. However, the overlay of differences in relative abundance across the network suggests that the Ruminococcaceae taxon specifically was more abundant in the Ruminococcaceae enterotype, while the opposite was true for the *Bacteroides* node. Consequently, there may be a link between CAN structure and enterotype assignment, which could be driven by stool moisture [27].

### The sponge CAN links to HMA–LMA status

We analyzed ten sponge order-specific networks that we inferred from Sponge Microbiome project data [31]. Due to their sessile lifestyle, sponges protect themselves from overgrowth, predation, and competition through production of bioactive compounds [34]. Such compounds may be produced by the sponges themselves or by their microbial symbionts [35]. Consequently, sponges may be expected to harbor symbiotic species that improve sponge health. While their open connection with their surroundings suggests that part of their microbiome may be transient, stable core microbiomes have been identified [36]. Therefore, our toolbox provides an opportunity to investigate conserved associations across sponges.

Networks were constructed with CoNet [2]. These networks had a median edge number of 137, with the smallest network containing 56 edges and the largest 1735 edges. We confirmed that a different network inference tool, FlashWeave, was able to recover many of the same associations despite large differences in network size (Additional File 1: Fig. S4) [7].

**Fig. 4:**
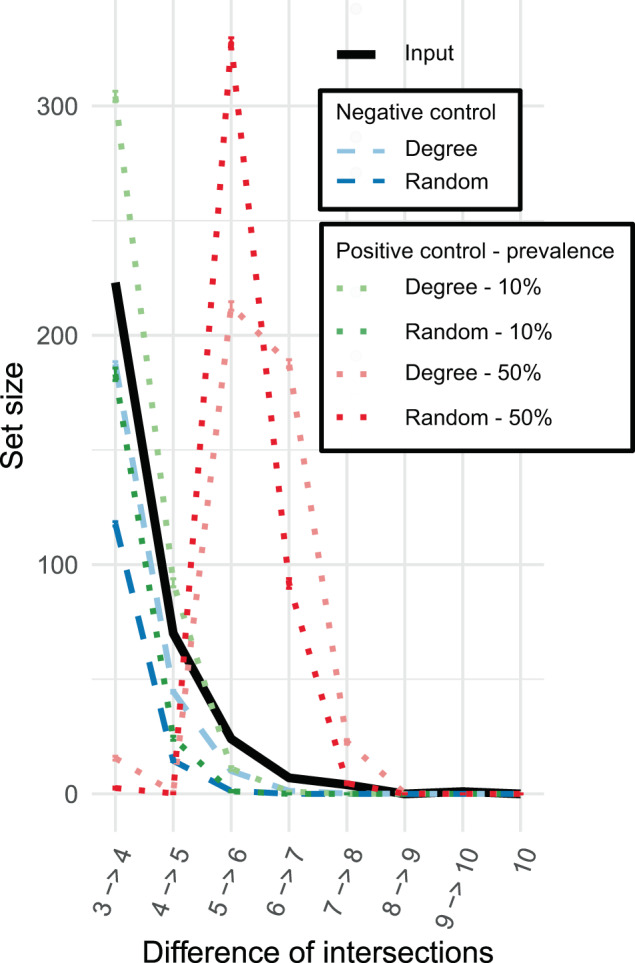
Set sizes across networks for ten sponge networks and randomizations of these networks. The set size is the number of edges present in a particular number of networks. The set size is shown for a set of sets of network intersections, meaning that the set of sets 4→6 is calculated as the number of edges in four or five networks with all edges in at least six networks removed. Each network was generated for a different host sponge order for which at least 50 samples were available. These networks were then randomized either with the same degree distribution (degree) or without this distribution (random). Moreover, each of the networks was randomized with preservation of a part of the input network for a subset of the randomizations as a positive control. Hence, the positive control degree networks are randomized versions of each input network with 20% of the union of associations present in at least 20% of observed networks or at least 50% of observed networks. Error bars represent the standard error across different combinations of random networks. For sets of edges present in up to six networks, the set size of the input networks deviates significantly from the set size from those of random networks with or without degree preservation.

**Fig. 5:**
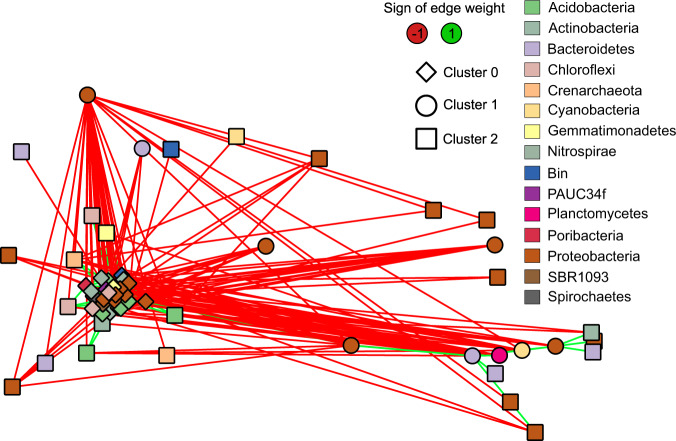
Core association network (CAN) constructed from associations present in at least three orders of sponges. Networks were generated with CoNet from samples collected for the Sponge Microbiome Project [31] and were constructed using samples from a single order of sponges. Associations present in at least three networks were included in the CAN. Node color was mapped to phylum and the manta clustering algorithm was used to identify clusters in the network.

Intersection differences up to six networks were significantly larger than differences generated from the randomized and degree-preserving negative control networks (*p* > 0.0001) (Fig. 4). However, the positive controls with a core conserved across 50% of networks had a much larger set size at five networks. Therefore, the CAN was constructed from all associations present in three out of ten networks (Fig. 5). Prior work suggests that most of the variation in a bipartite sponge-bacteria network could be attributed to differences between bacterial abundance: HMA versus LMA [31]. Supplementary data from Moitinho-Silva et al. [32] were used to identify taxa in network clusters that were significantly more or less abundant in HMA compared to LMA sponges. Indeed, we found that HMA and LMA assignments were different across the three clusters (Chi-squared test, *p* = 0.006), with cluster 0 containing more HMA-associated phyla and cluster 1 containing only LMA-associated phyla. This suggests that the CAN contains several phyla that have previously been identified as indicators of HMA–LMA status.

In addition to a CAN, we found that networks did not contain taxa with consistently lower or higher centrality scores compared to randomized networks (Additional File 1: Fig. S5).

## Discussion

Researchers can use properties of microbial association networks to describe trends in microbial communities. Such properties can include modularity, a CAN, high degree (hub nodes), or any other property that can be calculated from the network. Frequently, these are considered to mirror how the studied community is structured. Hence, robust estimates of these properties can yield valuable information on community structure.

We chose to use two types of network null models that represent two extremes in terms of constraints. The randomized null model is not constrained in terms of network structure (apart from node and edge number), while the degree-preserving models may be overly constrained especially if the degree centrality is a meaningful representation of a biologically relevant property, such as a taxon’s generalist lifestyle. Moreover, these models make no assumptions on the nature of associations. Therefore, it is unknown whether an association in a CAN reflects a biotic interaction, as these associations can also result from similar taxon responses to the environment or other organisms. The effects of biotic interactions can be better studied through other methods, for instance, joint species distribution models [37, 38].

Additional assumptions could be included to further improve the ability of null models to identify striking patterns. However, more complex null models are not well-established when it comes to the analysis of microbial association networks. Candidates include the Albert and Barabási [39] and Klemm and Eguíluz network models [40], which describe mechanisms of network growth and could therefore identify networks not generated in accordance with such mechanisms. Yet, a network model that assumes a particular growth mechanism may not be appropriate for association networks. Few associations in an association network are expected to represent interactions [41]. As a result, mechanisms for network growth may apply to the underlying interaction networks, but not directly to association networks. If a null model includes an assumption not known to be true, the null model becomes a pseudo-null model [42]. This could wrongly lead researchers to conclude that there is no relevant biological effect in addition to the effect described in the pseudo-null model. A comparison to these network models therefore addresses whether properties are significantly different compared to networks generated according to specific rules of network growth, but it cannot address the nonrandomness of network properties. Since this toolbox has been developed to find nonrandom trends in association networks, we chose network null models that make no assumptions on network growth (e.g., preferential attachment) because (1) we cannot be sure that such assumptions hold for interaction networks and (2) these null models do not take additional processes into account (such as environmental influence) that likely shape association networks.

As one of the null models in anuran preserves the degree distribution, comparisons to networks generated from those null models support statements on correlations between degree and other centralities. While we did not fully explore the effects of other properties, such as the fraction of realized edges or network topology, these topics have been discussed previously in methodological studies on ecological and social networks [15, 16, 43, 44]. However, we recommend that they deserve similar attention in the context of microbial association networks. For example, Agler et al. defined hub taxa as those taxa that had higher closeness, betweenness, and degree centrality than other taxa [45], but such measures may be strongly correlated in dissortative networks, where nodes with high degree are more likely to connect to nodes with low degree [46]. Our analysis of centrality rankings further supports the observation that betweenness and degree centrality are correlated, as we found that taxa did not have betweenness centrality rankings significantly different from betweenness centrality rankings observed for the degree-preserving random networks.

We found that our set-of-sets approach could identify a CAN from associations present in at least four individual-specific networks inferred from fecal samples. Out of the two largest CAN clusters, one contains *Blautia*, *Faecalibacterium*, and *Coprococcus* as its most central nodes, while the other contains *Sporobacter*, a group of Ruminococcaceae members, and a group of Clostridiales members. These clusters may be driven by enterotype structure. However, the number of individuals included in our analysis prevented us from drawing more specific conclusions.

On networks constructed from sponge order-specific taxon abundances, the set-of-sets approach identified a large CAN. This CAN was significantly different from the CAN observed for random networks. We suspected based on prior work that this large CAN could arise from a partition in sponge symbiotic relationships, as sponges tend to either have high or LMA [47]. Several taxa have been identified as indicators of this divide and many of those indicators were also found in the CAN [32]. Although HMA–LMA status is not strictly phylogenetically conserved across most sponges [47], associations between taxa that relate to this status appear to be conserved across at least a subset of sponge orders (Fig. 5). Hence, the set-of-sets analysis suggests that some of the dynamics responsible for the HMA–LMA discrepancy are shared across different orders of sponges.

No CAN was observed across 80–100% of samples. Either the associations exist and are not detected in several networks (false negatives) or associations are not highly conserved in these case studies. Our results support the latter explanation, since we detected traces of group structure that could have led to the low-prevalence CANs we observed. For the sponges, group-specific networks could be linked to HMA–LMA status [32]. For human individuals, group-specific networks could result from enterotype-specific variation in stool moisture, as moisture content was previously found to covary with stool samples that were enterotyped as *Prevotella* or *Bacteroides* [27]. However, due to the limited number of participants, we were unable to infer enterotype-specific CANs.

Microbial networks have become a popular method for the analysis of microbiome data despite their low accuracy. With anuran, we have introduced a tool that can aid in the comparison of multiple noisy networks through analysis of random networks. Our set-of-sets approach uses null models to find conserved patterns across groups of networks. Therefore, anuran is one of the first dedicated tools for meta-analysis of noisy networks with null models. We expect this null model suite to be a valuable benchmarking tool in the analysis of microbial and other networks.

## Supplementary information

Supplementary Figures

Supplementary File 1

## Data Availability

All scripts and software, including scripts to generate the figures in this manuscript, have been deposited to Zenodo [48]. An up-to-date version of the software is being maintained on a GitHub repository: https://github.com/ramellose/anuran [49]. Data for study case 1 will only be available upon acceptance of the corresponding manuscript. Data for study case 2 are available from [31].

## Materials availability

All scripts and software are available under the Apache 2.0 license.
