# Supplementary material for: Null-model-based network comparison reveals core associations
Source: ISME Commun. 2021 Jul 16;1:36. doi: 10.1038/s43705-021-00036-w (PMC9723671; doi:10.1038/s43705-021-00036-w)
Supplement: Supplementary file 1 — Supplementary Figures [file 43705_2021_36_MOESM1_ESM.pdf]

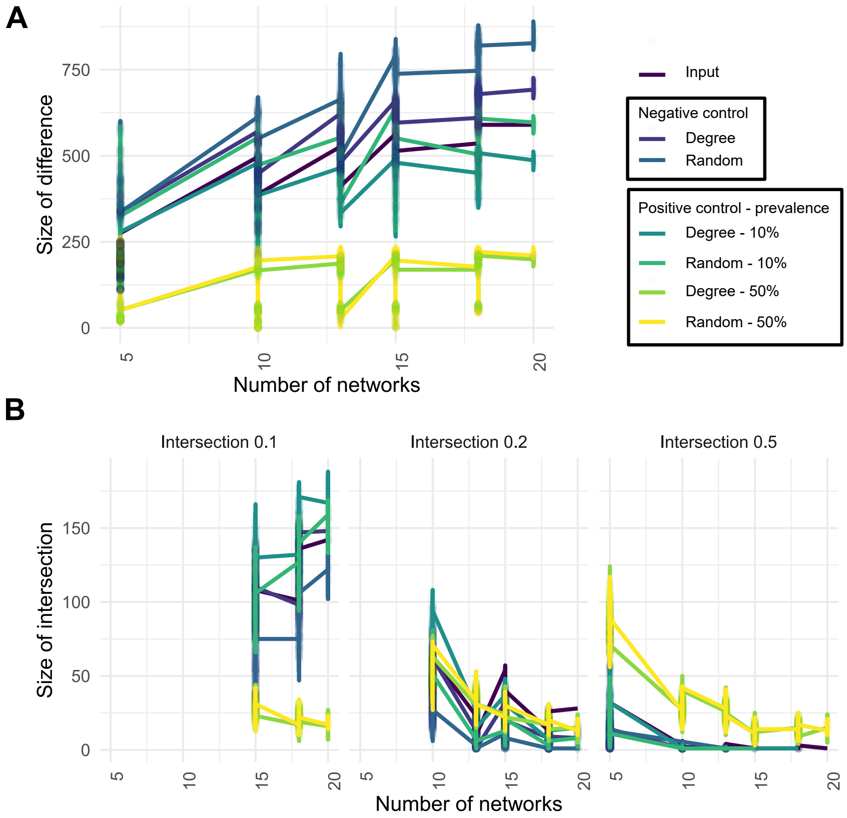

Figure S1: **Effect of the amount of gut microbiome networks on network intersections.** The set size is the number of edges present in a particular number of networks. The effect of sample number on set size was estimated by taking permutations of increasing numbers of networks. Each network was generated from stool samples collected from healthy volunteers and reflects a single volunteer. These networks were then randomized either with the same degree distribution (Degree) or without this distribution (Random). Both of these randomized networks were also generated with a number of fixed edges equal to 20% of the union of edges across networks, with edges fixed in 10% of networks or 50% of networks. **a** Effect of network amount on the difference, or the number of unique edges across all networks. **b** Effect of network amount on the intersection, or the the number of shared edges across a fraction of networks.

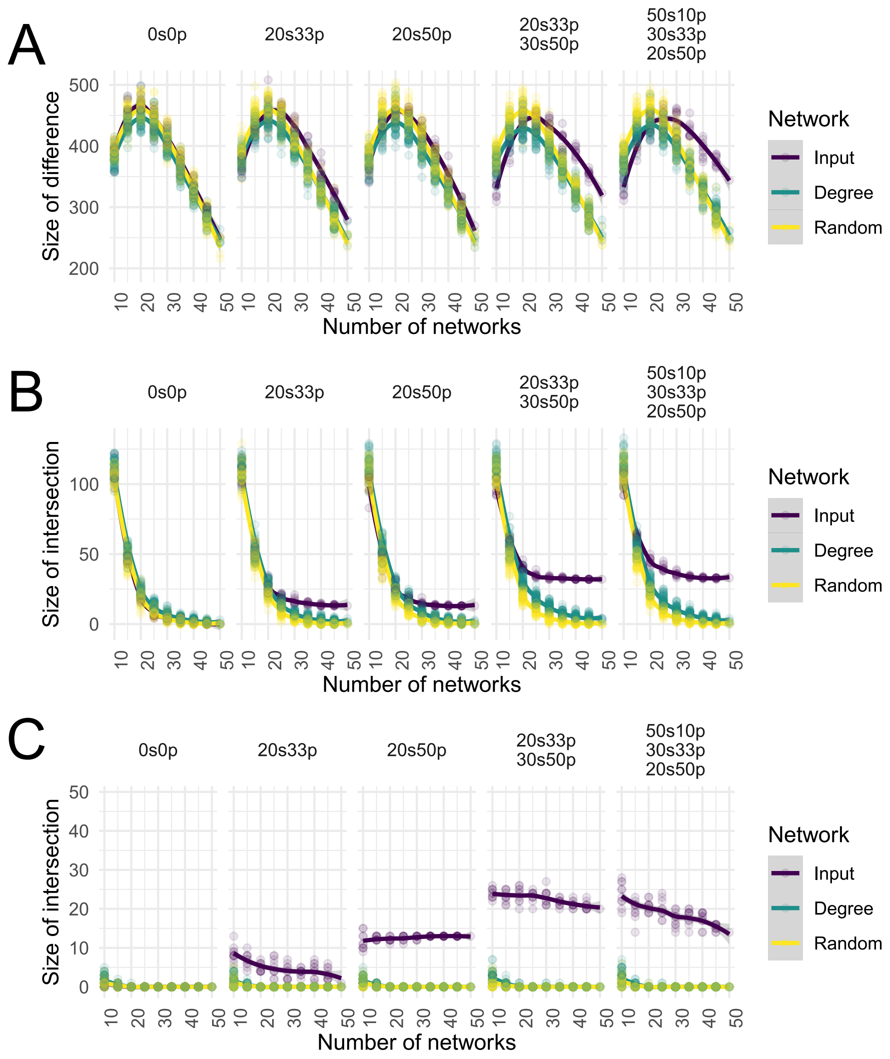

Figure S2: **Effect of the amount of simulated networks on network intersections.** The set size is the number of edges present in a particular number of networks. The effect of sample number on set size was estimated by taking permutations of increasing numbers of networks. These networks were generated with core networks of different sizes and prevalences. All networks contained 100 nodes. The shorthand above the figures refers to the core size as a percentage of network size (s) and core prevalence (p). For example, the figure titled 20s33p has a core network with a network size of 20% and prevalence of 33%. Cores do not overlap, so an edge present in the simulated 10% prevalence core is not part of the 33% prevalence core. These networks were then randomized either with the same degree distribution (Degree) or without this distribution (Random) with the specified fraction of edges kept fixed for a subset of networks. **a** Effect of sample numbers on the difference, the number of edges only present in a single network. **b** Effect of network number on the 20% intersection. **c** Effect of network number on the 40% intersection.

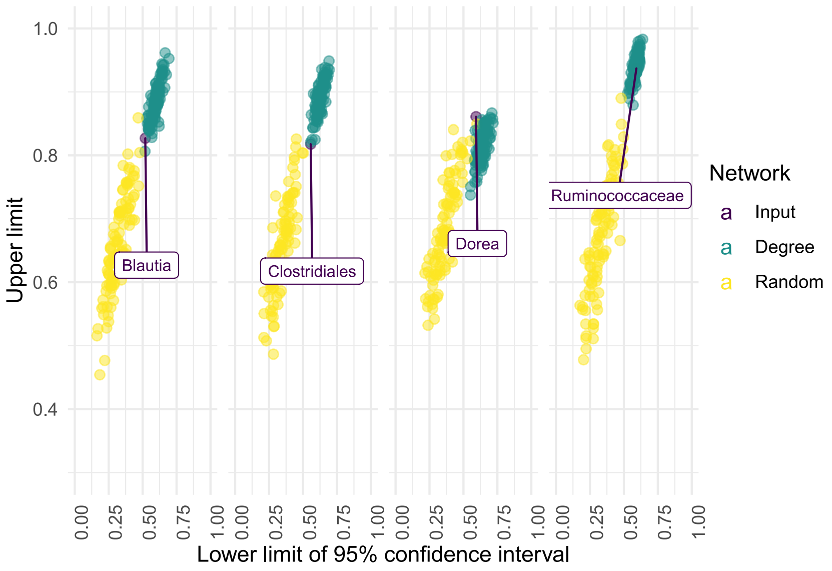

Figure S3: **Confidence intervals of betweenness centrality rankings for three taxa found in individual gut networks.** Estimated upper limit and lower limits for the 95% confidence intervals of betweenness centrality computed for each taxon across a group of networks and null models generated from these networks. Taxa that consistently have high centralities have narrower confidence intervals and are therefore located in the upper right of the plot, while taxa with consistent low centralities are located in the bottom right. Null models either had the same degree distribution (Degree) or were fully randomized (Random). The shown taxa were selected because they had a p-value below 0.15 for any of the permutation tests comparing closeness, betweenness or degree centrality rankings. The taxa assigned to the clades *Blautia*, *Clostridiales* and Ruminococcaceae had p-values of 0.119, 0.099 and 0.089 for the degree centrality test when compared to the fully randomized models, while *Dorea* had a p-value of 0.119 for the test comparing its closeness centrality to closeness centralities found in the randomized networks.

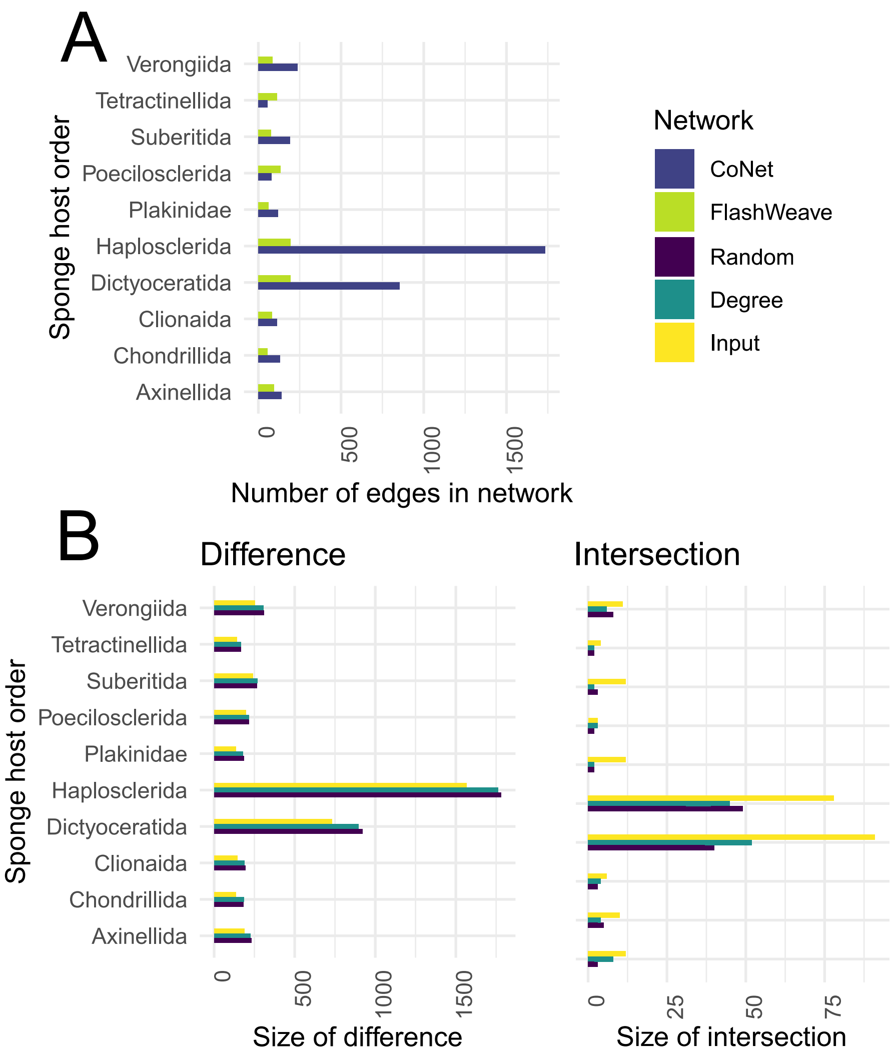

Figure S4: **Comparison of FlashWeave and CoNet networks on sponge order-specific networks.** Host order-specific networks were generated with both CoNet and Flashweave from samples collected for the Sponge Microbiome Project. We ran FlashWeave-S, so the samples were not considered to be heterogeneous. For the tool comparison, *anuran* generated five null models per order-specific network and resampled these five times. The labels on each figure refer to the sponge order. **a** Sizes of the FlashWeave and CoNet networks. **b** Difference and intersection of the FlashWeave and CoNet networks. The different bars represent the observed set size for the observed data (Input), the fully randomized networks (Random) and the degree-preserving randomized networks (Degree).

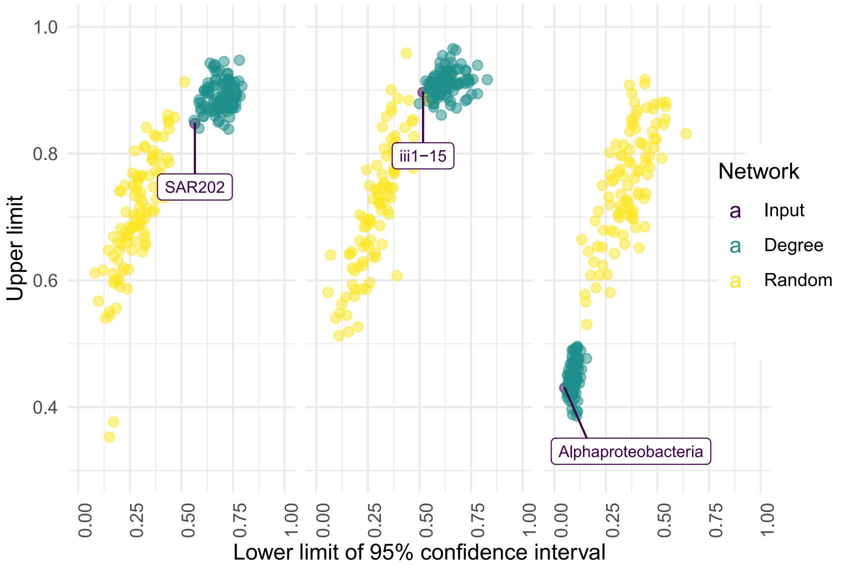

Figure S5: **Confidence intervals of betweenness centrality rankings for three taxa found in sponge order-specific networks.** Estimated upper limit and lower limits for the 95% confidence intervals of betweenness centrality computed for each taxon across a group of networks and null models generated from these networks. Taxa that consistently have high centralities have narrower confidence intervals and are therefore located in the upper right of the plot, while taxa with consistent low centralities are located in the bottom right. Null models either had the same degree distribution (Degree) or were fully randomized (Random). The shown taxa were selected because they had a p-value below 0.3 for any of the permutation tests comparing closeness, betweenness or degree centrality rankings. The taxa labelled SAR202, iii1-15 (a clade of Acidobacteria) and Alphaproteobacteria had p-values of 0.119, 0.188 and 0.129 for the degree centrality tests compared to the fully randomized models.
