# Supplementary material for: Null-model-based network comparison reveals core associations
Source: ISME Commun. 2021 Jul 16;1:36. doi: 10.1038/s43705-021-00036-w (PMC9723671; doi:10.1038/s43705-021-00036-w)
Supplement: Supplementary file 2 — Supplementary File 1 [file 43705_2021_36_MOESM2_ESM.pdf]

README.md

anuran 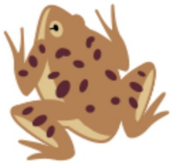

Toolbox for identification of non-random associations

build passing HitCount

This toolbox is intended to identify conserved or unique patterns across multiple networks. While carrying out set operations on such networks can help you find such associations, there is a chance that the outcome of the set operation was caused by random overlap between the networks. *anuran* helps you identify if your biological networks have set operations that have different outcomes than would be expected by chance. The simulated case study included with *anuran* can also help you design your experiments. Please find the anuran preprint on bioRxiv: https://doi.org/10.1101/2020.10.05.325860

Contact the author at lisa.rottjers (at) kuleuven.be. Your feedback is much appreciated! This version is still in development and has been tested for Python 3.6.

Installation instructions

You can use conda to install anuran. First add the channel hosting anuran and its dependencies:

```
conda config --add channels ramellose
```

Then create a new environment containing anuran:

```
conda create -n myenv anuran
conda activate myenv
```

You can then call the anuran command line tool from the conda environment.

Alternatively, to install *anuran* locally, run:

```
python3 -m pip install git+https://github.com/ramellose/anuran.git
```

anuran demo

To run the demo, run *anuran* as follows, with the output filepath changed to something suitable for your system. The software will generate several files, including anuran_demo_sets.csv, using the prefix specified in the command.

```
anuran -i demo -o output_filepath/anuran_demo -draw -perm 10 -nperm 10
```

The demo data for *anuran* was downloaded from the following publication:
Meyer, K. M., Memiaghe, H., Korte, L., Kenfack, D., Alonso, A., & Bohannan, B. J. (2018). Why do microbes exhibit weak biogeographic patterns?. The ISME journal, 12(6), 1404.
For a more elaborate demo analysis, please check out the vignette.

Manual

To run the script, only two arguments are required: input and output filepaths. The script recognizes gml, graphml and txt files (without headers) by their extension. The text files should be edge lists, with the third column containing edge weight.

```
anuran -i folder_with_networks -o filepath_to_output
```

You can specify more than one folder with the above parameter. If you want to compare across folders in addition to the null model comparison, add the flag below.

```
anuran -compare
```

anuran can also calculate gradients for ordered networks. If you have ordered networks, for example by constructing networks along a spatial or temporal gradient, *anuran* will test whether there is a correlation in network properties compared to the randomized networks. To use this feature, you need to use a naming convention for your networks. Within a folder, order the networks with a number and underscore, like below.

```
1_networkname.extension
2_networkname.extension
```

anuran generates randomized networks with permutations of the original network. By default, two types of randomized networks are generated: one that changes the degree distribution and one that does not. Note that the model changing the degree distribution may not have a major effect on the network structure as most smaller networks will not have enough dyad pairs to swap, especially if degree assortativity is large. You can specify the number of randomized networks with the parameter below.

```
anuran -perm              # number of randomized networks
```

It is possible to generate randomized networks with a specific core. Since we do not know the true core, the software generates a list of edges from the union of edges across all networks. A fraction of these edges is then included in randomized networks. For fully randomized networks, the core edges are added, then random edges are added until the original edge number is achieved. For the degree-preserving networks, edges in the network are first swapped. Then, the algorithm tries to find edges it can swap so the core edge is created without changing the degree of a node. This requires the nodes in the core edge to have another edge that is not part of the core. If those edges do not exist, it is not always possible to include a core edge while preserving node degree and a random edge is deleted instead to preserve edge number. For sparse or very small networks, the degree distribution may therefore change a little for positive control networks. If the core network is much larger than one of the observed networks, the degree distribution may also be affected.

If you want to simulate networks where a certain number of edges is conserved across networks, you can add the parameters below. Note that you can fill in more than one value. In this case, the cs parameter is the percentage of edges (of the total union of edges) that is conserved. The prev parameter defines the minimum number of networks, as a fraction of total networks, where these edges are found.

```
anuran -cs 0.3 0.5        # core size
anuran -prev 1            # core prevalence
anuran -gperm 10          # number of permutations
```

The set sizes are calculated for an intersection of 1 (all networks) by default. You can also choose to calculate the set sizes for all possible intersections of a number of the networks, i.e. each edge that was present on 2 out of 5 networks. If you flag the sign option, signs of edge weights are not taken into account. Normally, sets can have edges that have a unique edge sign in one network but a different edge sign in all others. If the sign option is flagged, this is not the case.

```
anuran -size 0.2 0.4 0.6                  # Calculates set sizes for edges present in partial intersections
anuran -sign                              # Ignores edge sign in set calculation
```

If you want to know how the set sizes change when you increase the number of replicates, use the parameter below; this will calculate set sizes for all numbers of networks up to the total number of networks. Up to 10 combinations are considered here.

```
anuran -sample 10
```

The sample size calculation can quickly become slow. If you are not interested in observing every single value, you can specify the sample numbers you are interested in.

```
anuran -n 5 10 20
```

In addition to set sizes, you can compute centrality scores (degree, betweenness and closeness centrality) and graph properties (assortativity, average shortest path length, connectivity, diameter and radius). If you want to know whether the set sizes, centralities or graph properties are different from randomized networks, you can run some statistics on these values. Note that the statistics are not reliable if you have fewer than 20 networks. **If you have fewer than 20 networks, some stats files may be empty and you will need to take the csv files and do statistical tests yourself.**

The centrality scores and graph properties are compared across networks using a Mann-Whitney *U*-test. The Mann-Whitney *U*-test does not require equal *n*; this is important since not all networks contain the same nodes. If there are too many unique nodes across networks, this test may give strange results. Hence. if you want to carry out these statistical tests, make sure that prevalence of most nodes is high across networks. For comparing graph properties, the p-value is computed using the standard score. This assumes that graph properties are normally distributed.

Note that calculations of centralities can take a long time, especially for large numbers of permutations. To compute centralities and graph properties and the associated test (with Bonferroni multiple-testing correction), add the following parameters:

```
anuran -c -net -stats bonferroni
```

For a complete explanation of all the parameters, run:

```
anuran -h
```

If the *stats* parameter is used, two files are exported for each requested measure: the file containing the original observations, and the file containing statistics for those observations. The statistics file may be empty for fewer than 20 networks but the calculations can then be done manually with the observations file. The columns refer to the network groups (Group, Network type) and specify null model settings (conserved fraction). For centrality, the upper and lower limits of the 95% confidence interval of the centralities is given in addition to the raw values. The set sizes include a 'Set size' column that includes the number of edges contained in a set, while the 'Set type' column specifies the set properties. The 'Set type (absolute)' column contains the value for the intersection converted to an absolute network number. Set differences are formatted similarly except the 'Set type' column is replaced by an 'Interval' column. Statistics files for these properties contain the 'Group' name column, the name of the group compared against 'Comparison', the type of observation that is being compared 'Measure', the p-value and the type of statistic that is used. The 'P.adj' column is the multiple testing-corrected p-value.

For documentation of specific functions, check out the Sphinx documentation.

Contributions

Any feedback or bug reports will be much appreciated! Please create an issue here to report any problems.

Authors

- **Lisa Röttjers** - ramellose
- **Karoline Faust** - hallucigenia-sparsa

See also the list of contributors who participated in this project.

License

This project is licensed under the Apache License - see the LICENSE.txt file for details
